# Proteome changes of dairy calves rumen epithelium from birth to postweaning

**DOI:** 10.3389/fgene.2022.1071873

**Published:** 2023-01-04

**Authors:** Kaizhi Zheng, Jianliang Wu, Saif Ullah, Yang Cao, Yongqing Jiang, Xin Huang, Junfang Jiang

**Affiliations:** ^1^ Institute of Animal Husbandry and Veterinary, Zhejiang Academy of Agricultural Sciences, Hangzhou, China; ^2^ Faculty of Veterinary and Animal Sciences, Lasbela University of Agriculture Water and Marine Sciences, Lasbela, Pakistan

**Keywords:** proteome, dairy calves, rumen, epithelium, postnatal development

## Abstract

**Background:** Rumen epithelium plays a central role in absorbing, transporting, and metabolizing of short-chain fatty acids. For dairy calves, the growth of rumen papillae greatly enhances the rumen surface area to absorb nutrients. However, the molecular mechanism underlying dairy calves rumen postnatal development remains rarely understood.

**Results:** Here, we firstly describe the histological change of rumen epithelium from birth to day 90 of age. Then, a shotgun approach and bioinformatics analyses were used to investigate and compare proteomic profiles of Holstein calve rumen epithelium on day 0, 30, 60 and 90 of age. A total of 4372 proteins were identified, in which we found 852, 342, 164 and 95 differentially expressed proteins between D0 and D30, between D30 and D60, between D60 and D90, respectively. Finally, Gene Ontology and Kyoto Encyclopedia of Genes and Genomes (KEGG) analyses were performed to provide a comprehensive proteomic landscape of dairy calves rumen development at tissue level.

**Conclusion:** To conclude, our data indicated that keratinocyte differentiation, mitochondrion formation, the establishment of urea transport and innate immune system play central roles during rumen epithelium development. Tetrahydrobiopterin (BH4) presents an important role in rumen epithelial keratinization. The biological processes of BH4 biosynthesis and molecular function of nicotinamide adenine dinucleotide phosphate binding participate in mitochondrial cristae formation. The proposed datasets provide a useful basis for future studies to better comprehend dairy calves rumen epithelial development.

## 1 Introduction

Ruminants possess remarkable and distinct multi-chambered stomachs that allows them to utilize plant fiber as energy source. The consumed plant fiber is degraded by diverse microbiota in the rumen into precursors of essential metabolites and metabolic cofactors (Xu et al., 2021). For dairy calves, replacement heifers are the second largest annual operating expense on the farm, which is critical for the future of dairy farms in improving genetic merit and maintain herd size (Tozer and Heinrichs, 2001). During replacement heifer stage, the growth of rumen papillae greatly enhances the rumen surface area to absorb nutrients, while the development of the rumen epithelium is a critical physiological challenge to transit from solid diet to milk (Baldwin and Connor, 2017; Diao et al., 2019). Rumen epithelium plays a central role in absorbing, transporting, and metabolizing of short-chain fatty acids (SCFAs) after the rumen microbes digest fiber (Ji et al., 2021). SCFAs can provide up to 70% of the energy requirements of ruminants (Bergman, 1990). Thus, a well-developed rumen is essential for the raise of replacement heifer, which will influence the performance of dairy cow in future.

Unlike the monogastric stomachs, the rumen tissue owns a complex structure consisting of stratified squamous epithelium, lamina propria, mucosa, tunica muscularis and plasma membrane (Baldwin, 1998). Thus, the postnatal development of rumen epithelium differs from that of monogastric animals. After birth, dramatic structural and functional changes occur during postnatal rumen development, in which rumen papillae grow and the SCFAs metabolism machinery gradually matures (Xu et al., 2022). However, current understanding to the biological foundation of rumen epithelium development remains limited. Hence, an overview profile of protein expression during rumen epithelium development is essential to understand its biological foundation (Finnegan et al., 2019). Nowadays, proteomics, as a powerful tool, is widely applied to identify and quantify overall proteins and present content of a cell, tissue or an organism (Aslam et al., 2017). Given the central role of rumen epithelium in dairy calves production, we were promoted to use proteomic as a powerful tool to illuminate the underlying molecular mechanisms that involved in rumen epithelium postnatal development.

## 2 Material and methods

### 2.1 Animals

The male Holstein calves at the age of day 0, day 30, day 60 and day 90 were randomly selected with 5 calves in each age. Rumen epithelia were isolated and frozen in liquid nitrogen. Experimental protocols for animal research were approved by the Institutional Animal Care and Use Committees at the Zhejiang Academy of Agricultural Sciences.

### 2.2 Histology analysis

The histological analysis was performed essentially as the procedure described previously (Zheng et al., 2021), the rumen tissues kept in PFA were dehydrated with alcohol and embedded in paraffin, sectioned at 5 μm, and stained with hematoxylin and eosin (H&E), and the sections were observed under a microscope (Nikon, NY, United States).

### 2.3 Sample preparation and trypsin digestion for label-free proteome

The sample preparation of protein was performed essentially as the procedure described previously (Zheng et al., 2022). Rumen epithelium samples were ground individually in liquid nitrogen and lysed with PASP lysis buffer (100 mM NH_4_HCO_3_, 8 M Urea, pH 8.0), followed by 5 min of ultrasonication on ice. The lysate was centrifuged at 12000 g for 15 min at 4°C. The supernatants were collected and quantified by BCA Protein Assay Kit (Beyotime Institute of Biotechnology, Shanghai, China). 20 μg of the protein sample was loaded to 12% SDS-PAGE gel electrophoresis for quality control and the supernatant was reduced with 10 mM DTT for 1 h at 56°C, and subsequently alkylated with sufficient iodoacetamide for 1 h at room temperature in the dark. Then samples were completely mixed with 4 times volume of precooled acetone by vortexing and incubated at −20°C for at least 2 h. Samples were then centrifuged at 12000 g for 15 min at 4°C and the precipitation was collected.

After washing with 1 ml cold acetone, the pellet was dissolved by dissolution buffer (8 M Urea, 100 mM TEAB, pH 8.5). Trypsin and 100 mM TEAB buffer were added, sample was mixed and digested at 37°C for 4 h. Then trypsin and CaCl_2_ were added digested overnight. Formic acid was mixed with digested sample, adjusted pH under 3, and centrifuged at 12000 g for 5 min at room temperature. The supernatant was slowly loaded to the C18 desalting column, washed with washing buffer (0.1% formic acid, 3% acetonitrile) 3 times, then added elution buffer (0.1% formic acid, 70% acetonitrile). The eluents of each sample were collected and lyophilized.

### 2.4 LC-MS/MS analysis

LC-MS/MS analysis was then performed according to the procedure described previously (Zhou C. Y. et al., 2019). Mobile phase A (0.1% formic acid in H_2_O) and B solution (0.1% formic acid in 80% acetonitrile) were prepared. The lyophilized powder was dissolved in 10 μL of solution A, centrifuged at 14000 g for 20 min at 4°C, and 1 μg of the supernatant was injected into a home-made C18 Nano-Trap column (4.5 cm × 75 μm, 3 μm). Peptides were separated in a home-made analytical column (15 cm × 150 μm, 1.9 μm), using a linear gradient elution.

LC-MS/MS analyses were performed by using Q ExactiveTM series mass spectrometer (Thermo Fisher, Germany) at Novogene Co., Ltd. (Beijing, China), with ion source of Nanospray Flex™ (ESI), spray voltage of 2.1 kV and ion transport capillary temperature of 320°C. Full scan range from m/z 350 to 1500 with resolution of 60000 (at m/z 200), an automatic gain control target value was 3 × 10^6^ and a maximum ion injection time was 20 ms. The top 40 precursors of the highest abundant in the full scan were selected and fragmented by higher energy collisional dissociation and analyzed in MS/MS, where resolution was 15000 (at m/z 200), the automatic gain control target value was 1 × 10^5^, the maximum ion injection time was 45 ms, a normalized collision energy was set as 27%, an intensity threshold was 2.2 × 10^4^, and the dynamic exclusion parameter was 20 s. The raw data of MS detection was named as “.raw”.

### 2.5 Protein identification and quantitation

The all resulting spectra were searched by Proteome Discoverer 2.2 (PD 2.2, Thermo). The corresponding proteins were matched with *bovine* database. The search parameters are set as follows: mass tolerance for precursor ion was 10 ppm and mass tolerance for product ion was 0.02 Da. Carbamidomethyl was specified as fixed modifications, Oxidation of methionine was specified as dynamic modification, and acetylation was specified as N-Terminal modification. A maximum of two missed cleavage sites were allowed. To improve the quality of analysis results, the software PD 2.2 further filtered the retrieval results: Peptide Spectrum Matches (PSMs) with a credibility of more than 99% was identified PSMs. The identified protein contains at least 1 unique peptide. The identified PSMs and protein were retained and performed with FDR no more than 1.0%. Principal component analysis (PCA) and volcano plot, which combined fold-change analysis and t-tests, were performed. Proteins with a minimum fold change of 2 (ratio >2 or <0.5, *p* < 0.05) were considered to be regulated differently between comparisons.

### 2.6 Bioinformatics analysis

GO functional analysis was conducted using the interproscan program against the non-redundant protein database (including Pfam, PRINTS, ProDom, SMART, ProSite, PANTHER), and the databases of COG (Clusters of Orthologous Groups) and KEGG (http://www.genome.jp/kegg/) were used to analyze the protein family and pathway. DPEs were used for Volcanic map analysis, cluster heat map analysis and enrichment analysis of GO, subcellular localization and KEGG. The probable protein-protein interactions were predicted using the STRING-db server (http://string.embl.de/).

## 3 Results

### 3.1 The histological changes of ruminal papillae during postnatal stage

To have an overview of rumen epithelium postnatal development, we firstly evaluated the histological changes of rumen papillae from birth to 90 days old. The rumen epithelium experienced dramatic transformation to tongue shape papillae on day 30 of age. Thereafter, the papilla underwent a period of growth until 90 days of age.

**FIGURE 1:**
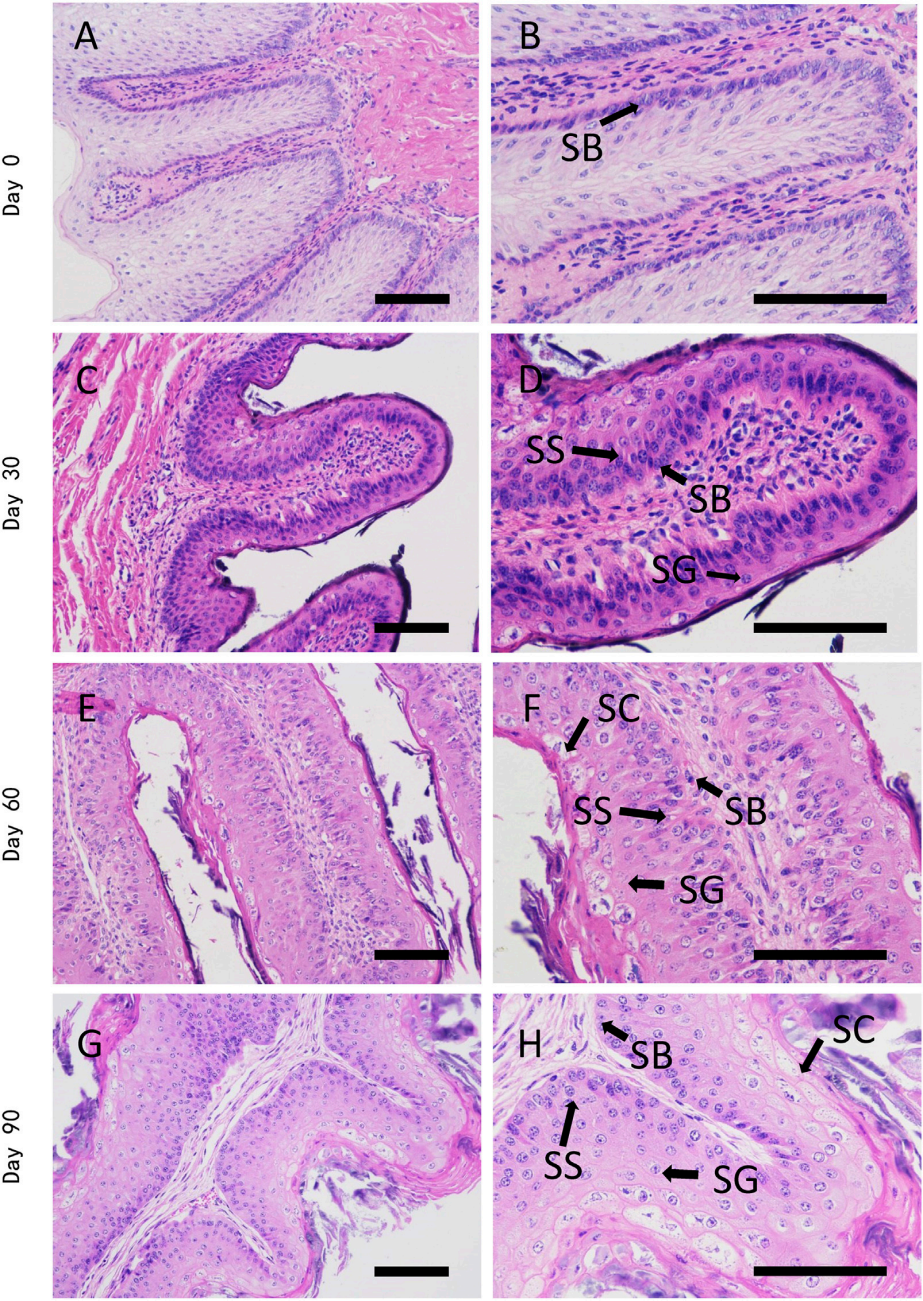
H&E staining of dairy cattle rumen epithelium on the age of day 0 **(A**,**B)**, day 30 **(C**,**D)**, day 60 **(E**,**F)**, and day 90 **(G**,**H)**. SB is stratum basale, SS is stratum spinosum, SG is stratum granulosum, SC is stratum corneum. Bar is 50 μm.

On the first day of Holstein diary calve postnatal life, the papillae were formed by a single layer of stratum basale cells that covered papillary core. The papillary core was covered by a few layers of stratified squamous cells, which were covered by serosa. The rumen epithelia then formed tongue-shaped papillae on day 30 of age, which were constituted by stratum basale, stratum spinosum and stratum granulosum. On 60 days of age, the space between papillae was larger than that of day 30, and stratum corneum could already be clearly observed. We could also found branch like morphology on tongue-shaped papillae since this stage. On 90 days of age, the stratum corneum covered most of the papillae except the top of papillae. In spite of more layers of stratified squamous cells, we didn’t found significant histological changes of ruminal papillae on 90 days of age, when comparing with day 60 (Figure 1).

### 3.2 Protein identification and comparison analysis

**FIGURE 2:**
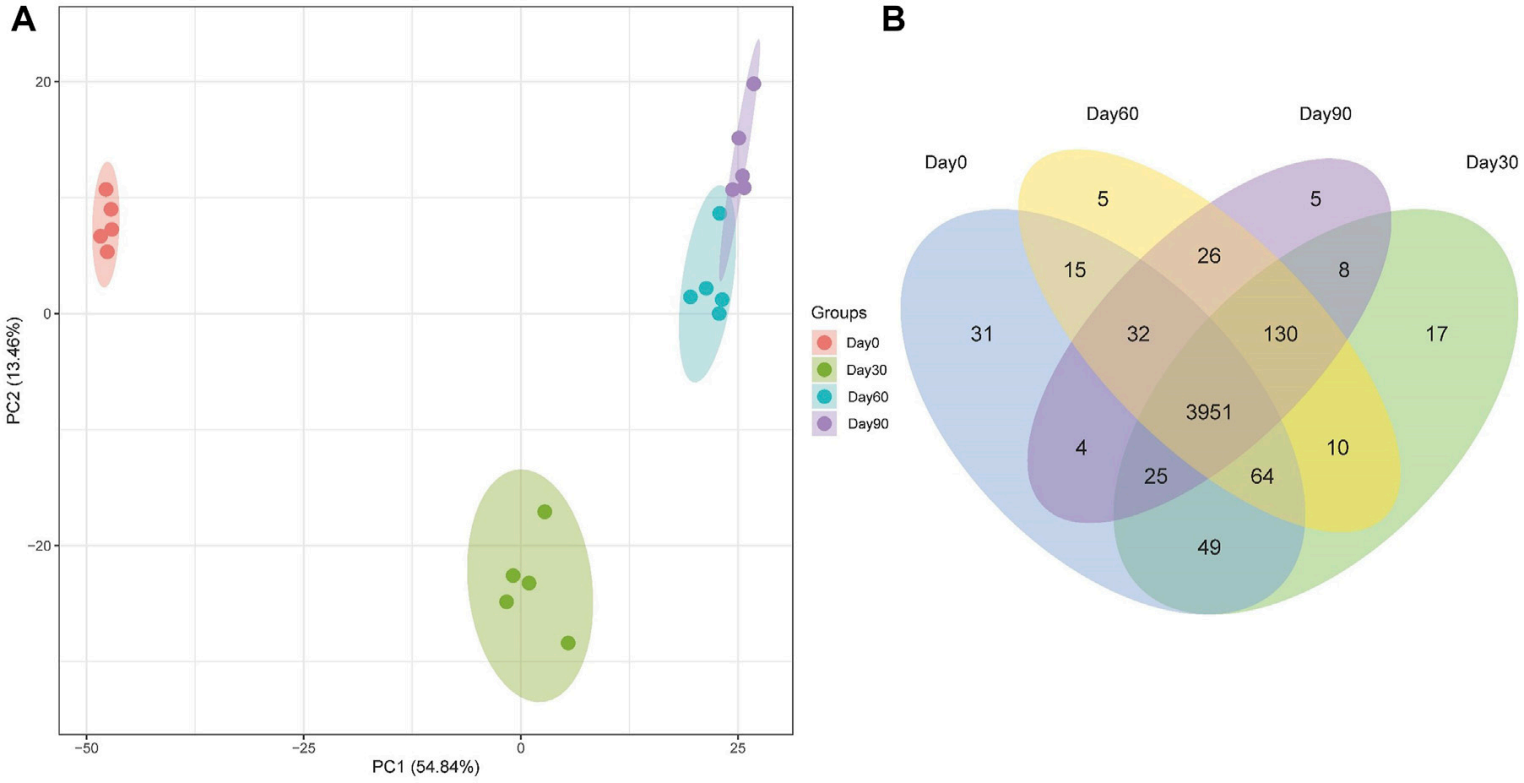
PCA scores plot **(A)** and Venn Diagram **(B)** of proteins identified in rumen epithelium at the age of day 0, day 30, day 60, and day 90.

To explore the mechanism driving Holstein calves rumen papillae development, label-free proteomic strategy was used to identify different abundant proteins of day 0, day 30, day 60 and day 90. A total of 4372 proteins were identified, including 3951 shared proteins, 31 proteins uniquely expressed in day 0, 17 proteins uniquely expressed in day 30, 5 proteins uniquely expressed in day 60 and day 90 (Figure 2A). PCA analysis was performed to visually differentiate the sample clusters among the observations. We found that 68.30% of the variability was explained by the first two principal components, which accounted for 54.84%, and 13.46% of the total variance. The rumen epithelium of day 0, day 30 and day 90 could be separated completely by identified proteins and distributed in different locations. However, rumen epithelium of day 60 could not be completely separated from that of day 30 and day 90 (Figure 2B).

**FIGURE 3:**
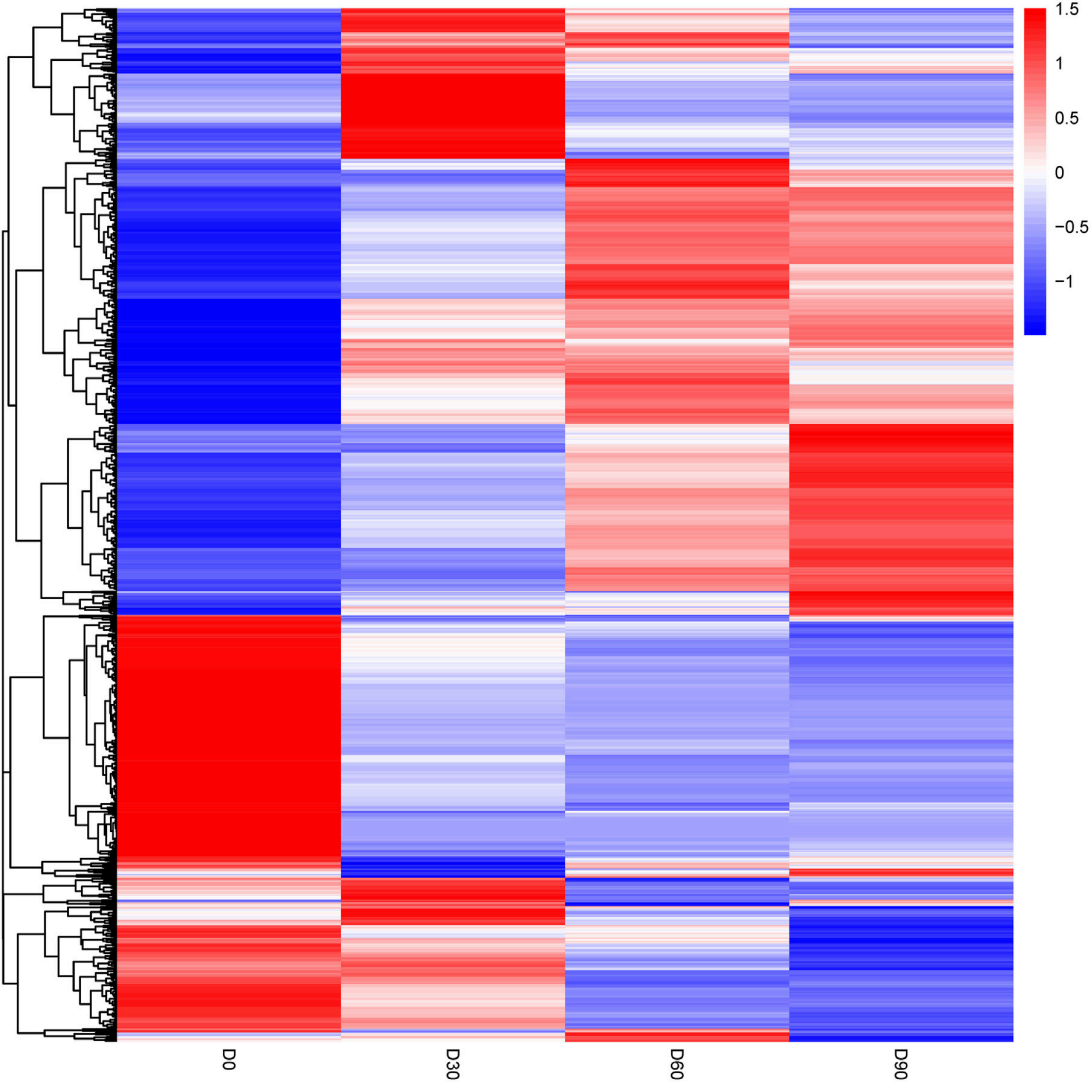
Heat map of differentially abundant proteins in rumen epithelium at the age of day 0, day 30, day 60 and day 90.

The differentially abundant proteins were highlighted by simultaneously considering fold change >2 or <0.5 and *p* < 0.05. A total of 452 proteins were significantly different in comparison of day 0 and day 30 rumen epithelium (Supplementary Table S1). While the number of DEPs was 152 between rumen epithelium of day 30 and day 60 (Supplementary Table S2). And finally, this number became 34 between day 60 and day 90 (Supplementary Table S3). Unsupervised hierarchical clustering of different biological data sets was then conducted as a more rigorous test for the screened proteins to evaluate the rationality, accuracy and dynamic changes of these DEPs (Figure 3).

### 3.3 Bioinformatics analysis of differentially abundant proteins

**FIGURE 4:**
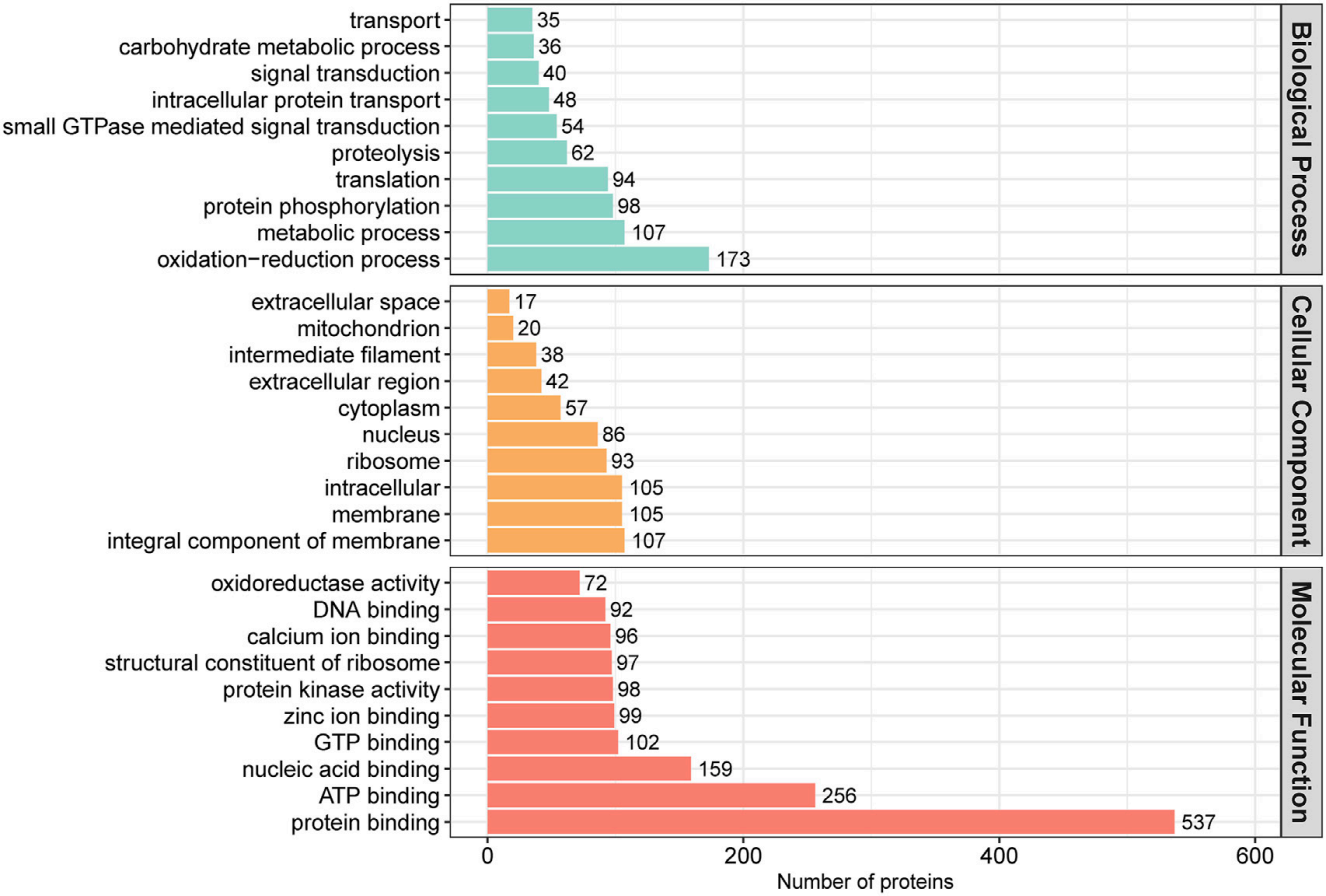
GO annotation classification of differentially abundant proteins in rumen epithelium at the age of day 0, day 30, day 60 and day 90.

**FIGURE 5:**
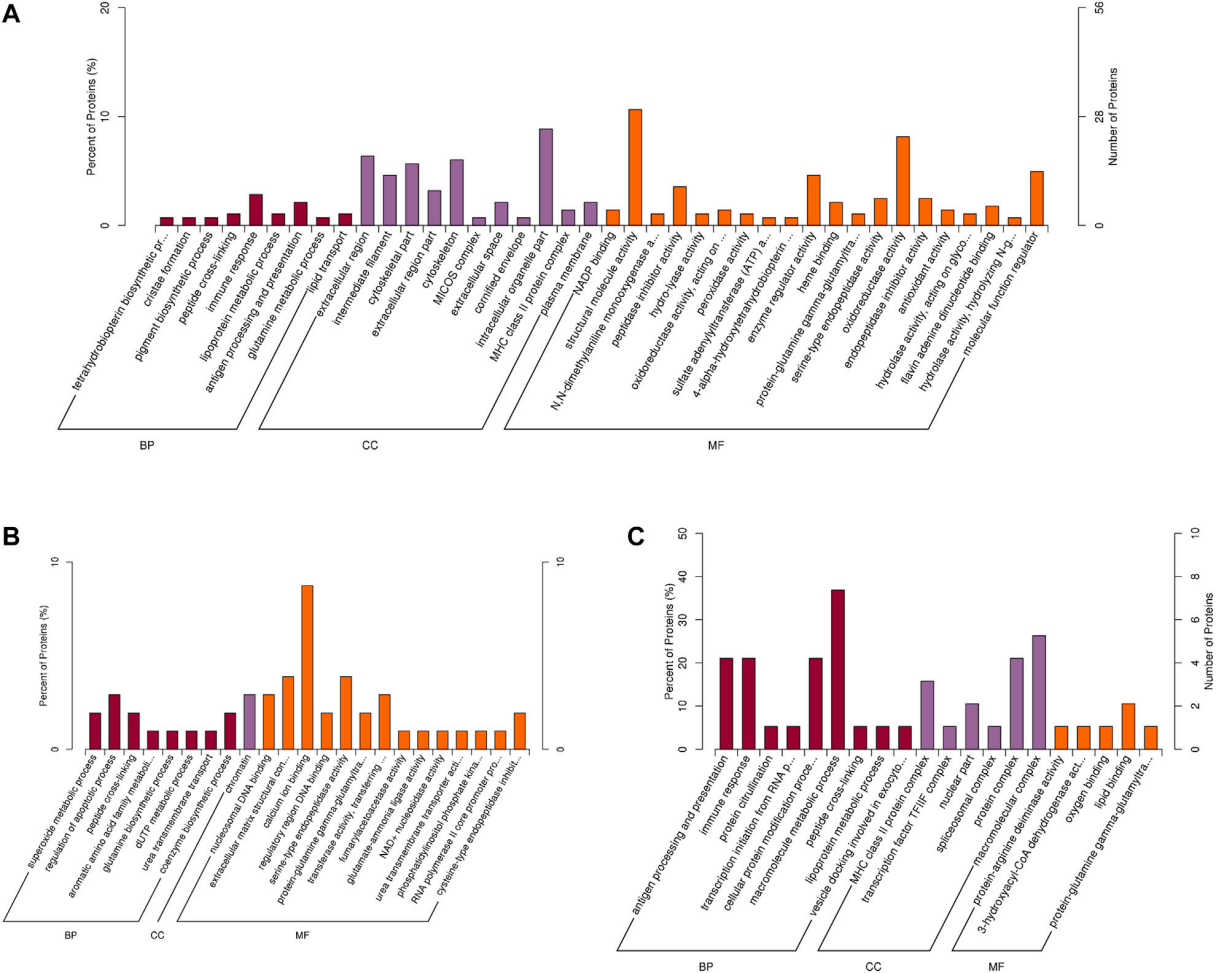
GO enrichment analysis of differentially abundant proteins in rumen epithelium between **(A)** day 0 and day 30, **(B)** day 30 and day 60, and **(C)** day 60 and day 90 of age. All pathways are listed according to *p*-value.

In order to assess the major biological processes that participated in the postnatal development of calves rumen epithelia, a GO annotation was performed to analyze the functional characteristics of proteins identified in calves rumen epithelia of day 0, day 30, day 60 and day 90 (Figure 4). Bioinformatics analyses were then performed to construct a specific molecular network to explore the biological functions and pathways related to the DEPs from birth to day 90 of age. GO enrichment analysis showed that DEPs in rumen epithelia were significantly enriched in 41 GO terms between day 0 and day 30 (*p* < 0.05). In the biological processes analysis, the top five biological progresses were tetrahydrobiopterin biosynthetic process (GO:0006729), cristae formation (GO:0042407), pigment biosynthetic process (GO:0046148), peptide cross-linking (GO:0018149) and immune response (GO:0006955). In molecular functions, the top five significant GO terms were NADP binding (GO:0050661), structural molecule activity (GO:0005198), N,N-dimethylaniline monooxygenase activity (GO:0004499), peptidase inhibitor activity (GO:0030414) and hydro-lyase activity (GO:0016836). As for cell components, extracellular region (GO:0005576), intermediate filament (GO:0005882), cytoskeletal part (GO:0044430), extracellular region part (GO:0044421) and cytoskeleton (GO:0005856) were the top five GO terms that enriched significantly (*p* < 0.05) (Figure 5A, Supplementary Table S4).

While the number of GO enriched terms decreased to 23 between day 30 and day 60 of age. The top five molecular function GO terms were nucleosomal DNA binding (GO:0031492), extracellular matrix structural constituent (GO:0005201), calcium ion binding (GO:0005509), regulatory region DNA binding (GO:0000975) and serine-type endopeptidase activity (GO:0004252). As for biology processes, superoxide metabolic process (GO:0006801), regulation of apoptotic process (GO:0042981), peptide cross-linking (GO:0018149), aromatic amino acid family metabolic process (GO:0009072) and glutamine biosynthetic process (GO:0006542) were the top five significantly enriched GO terms. And for cell components, the significantly enriched GO term was chromatin (GO:0000785) (*p* < 0.05) (Figure 5B, Supplementary Table S5).

Between rumen epithelium tissues of day 60 and day 90, the significantly enriched GO terms of biology progresses were antigen processing and presentation (GO:0019882), immune response (GO:0006955), protein citrullination (GO:0018101), transcription initiation from RNA polymerase II promoter (GO:0006367), cellular protein modification process (GO:0006464), macromolecule metabolic process (GO:0043170). As for molecular functions, the GO terms included protein-arginine deiminase activity (GO:0004668), 3-hydroxyacyl-CoA dehydrogenase activity (GO:0003857), lipid binding (GO:0008289) and protein-glutamine gamma-glutamyltransferase activity (GO:0003810). In cell components, DEPs were significantly enriched in MHC class II protein complex (GO:0042613), transcription factor TFIIF complex (GO:0005674), nuclear part (GO:0044428) spliceosomal complex (GO:0005681) and protein complex (GO:0043234) (*p* < 0.05) (Figure 5C, Supplementary Table S6).

### 3.4 KEGG pathway enrichment analysis

**FIGURE 6:**
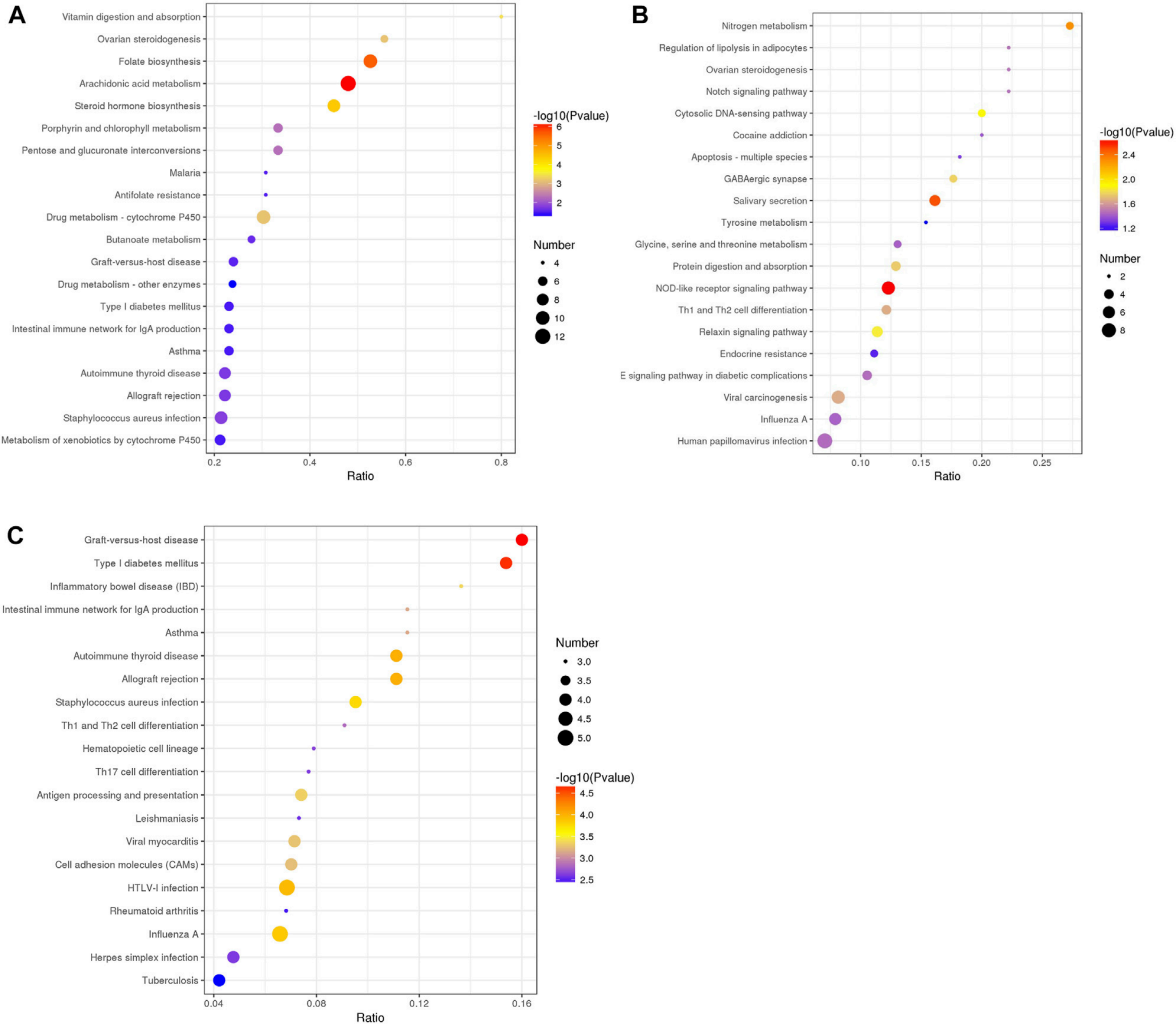
KEGG pathway enrichment analysis of differentially abundant proteins in rumen epithelium between **(A)** day 0 and day 30, **(B)** day 30 and day 60, and **(C)** day 60 and day 90 of age. All pathways are listed according to *p*-value.

Then, we performed KEGG pathway enrichment analysis to extract the biological pathways related to the DEPs in rumen epithelia from birth to day 90 of age. Between day 0 and day 30, the DEPs were significantly enriched in 24 pathways. The top five significantly enriched KEGG terms were Arachidonic acid metabolism (map00590), Folate biosynthesis (map00790), Steroid hormone biosynthesis (map00140), Vitamin digestion and absorption (map04977), Drug metabolism - cytochrome P450 (map00982) (*p* < 0.05) (Figure 6A, Supplementary Table S7). From day 30 to day 60 of age, the top five significantly enriched KEGG terms were NOD-like receptor signaling pathway (map04621), Salivary secretion (map04970), Nitrogen metabolism (map00910), Cytosolic DNA-sensing pathway (map04623) and Relaxin signaling pathway (map04926) (*p* < 0.05) (Figure 6B, Supplementary Table S8). From day 60 to day 90 of age, the top five significantly enriched KEGG terms were Graft-versus-host disease (map05332), Type I diabetes mellitus (map04940), Autoimmune thyroid disease (map05320), Allograft rejection (map05330), HTLV-I infection (map05166) (*p* < 0.05) (Figure 6C, Supplementary Table S9).

## 4 Discussion

To the best of our knowledge, the present study is the first to systemically characterize proteome changes of diary calve rumen epithelium from birth to day 90 of age. Our research also provides extensive and functional analyses of diary calve rumen epithelium, which provides a useful basis to better comprehend cow rumen epithelial development. Rumen is an essential organ in ruminants, which plays principal roles in nutrient metabolism and transportation. The development of rumen epithelium to tongue-shaped papillae increases the surface area of rumen wall, which contributes to the absorption of SCFAs (Nishihara et al., 2019). During dairy cow postpartum period, a well-developed papilla can promote its absorption rate, which increases the dry matter intake and milk production (Miller et al., 2021). Hence, the development of rumen epithelium at early stage determines the future production of milk. From monogastric animal to a ruminant, the dairy calves undergoes a series of dramatic gastrointestinal transformations after birth, which changes from a milk-based to a solid-based diet (Steele et al., 2016; Meale et al., 2017). Thus, understanding the mechanism that drives the transformation of cow rumen epithelium is important for devising strategies to improve ruminant productivity (Roh et al., 2016). However, the molecular mechanism regulating the growth of rumen epithelium remains unclear. In this study, the histological and morphological changes of calves rumen epithelium were described. We also analyzed the proteomic changes of 20 rumen tissue samples during four stages of dairy calves, which provided a comprehensive landscape dairy calves rumen epithelium from birth to day 90 of age.

The high consistence of PCA and heat map analysis results showed that the DEPs upon different ages were distinguishable among rumen epithelium samples of day 0, day 30 and day 60. While the results of day 60 could not be distinguished from that of day 90. These data indicate that significant histological changes occur from day 0 to day 30 and from day 30 to day 90.

The rumen epithelium experienced a dramatic transformation from a single layer of stratum basale cells covered by a few layers of stratified squamous cells to tongue-shaped papilla constituted by stratum basale, stratum spinosum and stratum granulosum. Stratum corneum is the outermost barrier of rumen epithelium that prevent the infection of rumen microorganisms. The keratinization of stratified squamous epithelium of the rumen is similar to that of skin epidermis (Graham and Simmons, 2005). Located at the rumen epithelial surface, keratinocytes are constantly exposed to external stimuli and are the first barrier to invading pathogens (Coulombe, 2017). In keratinocytes, keratins are the major structural intermediate filament proteins, which are expressed in a highly specific pattern at different differentiation stages of keratinocytes. Thus, it is widely used as biomarkers for identifying the stage of epithelial cell proliferation or differentiation (Zhang et al., 2019). Previous studies have suggested that the KRT4 gene and keratinocyte differentiation pathway play key roles in stratum corneum formation by transcriptomic analyses (Bragulla and Homberger, 2009). A recent study has also identified KRT17 and KRT15 as main keratin-encoding gene in two different stages of rumen epithelium keratinization in sheep (Yuan et al., 2022). Consistent with previous studies, we found the protein expression of keratin 17 down-regulated on day 0 of age, while the expression of keratin 15 and keratin 4 gradually decreased from day 0 to day 60 of age. These results indicated that keratinocyte differentiation gradually decreased from day 0 to day 60 of age. We also found keratin 6 up-regulated and keratin 7, keratin 80, keratin 85, keratin 75 and keratin 72 down-regulated on day 30 of age, when comparing with that of day 0. Among these keratins, keratin 6 regulates collective keratinocyte migration (Wang et al., 2018). Keratin 7 is a type II intermediate filament protein which is primarily expressed in simple-type epithelia of glandular and ductal cells, as well as in stratified and transitional epithelia of urothelial and mesothelial tissues (Alam et al., 2021). Keratin 85 and keratin 75 are expressed primarily in hair follicles, nail beds, and lingual papillae (Yang et al., 2019). Keratin 80 facilitates the viability, proliferation, and migration of gastric cancer cells and inhibits cell apoptosis (Zhang et al., 2022). Thus, the role of these keratin proteins in rumen epithelium remains to be explored.

BH4 functions as a cofactor to convert amino acids to precursors of dopamine and serotonin (Li et al., 2015). In this study, we found DEPs significantly enriched in BH4 biosynthetic process, and pterin-4-alpha-carbinolamine dehydratase (PCD) and 4a-hydroxytetrahydrobiopterin dehydratase (DH) up-regulated on day 0 of age. PCD is required for enzymatic regeneration of BH4 (Citron et al., 1992). While, DH is the rate-limiting enzyme for the recycling of BH4, and its activity significantly decreased in differentiated keratinocytes (Schallreuter et al., 1995). Thus, our data indicated that rumen epithelium of day 30 might contain more differentiated keratinocytes, while BH4 presents an important role in rumen epithelial keratinization.

The generation of BH4 is mediated by nicotinamide adenine dinucleotide (NADH) (Werner et al., 2011). In mitochondrion, BH4 is critical for maintaining its proper morphology (Zhou J. et al., 2019). Mitochondrion is a complex organelle that is essential in energy transduction and cellular signaling events. In rumen epithelium, mitochondria are majorly located in stratum basale cell (Graham and Simmons, 2005), which is considered to play a crucial role in the metabolism of SCFAs (Leighton et al., 1983). While the integrality of mitochondria morphology is also essential for cell differentiation, since it is usually accompanied by the activation of mitochondrial respiration, in which mitochondria exhibit a shift from glycolysis to oxidative phosphorylation (OXPHOS) (Shyh-Chang et al., 2013). The proton gradient across the cristae of mitochondria is the basis of OXPHOS, which provides the energy for cellular differentiation and proliferation (Ikon and Ryan, 2017). In this study, we found DEPs between rumen epithelium of day 0 and day 30 significantly enriched in molecular function of NADP binding, biological process of BH4 biosynthetic process and cristae formation. The proteins of mitochondrial part significantly up-regulated in day 30 of age, when the expression of mitochondrial contact site and cristae organizing system (MICOS) complex subunit and MICOS complex subunit MIC27 increased on day 30 of age. These data indicated an important role of mitochondrion during early calves rumen development, in which the biological processes of BH4 biosynthesis and molecular function of NADP binding participate in mitochondrial cristae formation.

Rumen epithelium, as the outermost barrier of calves, is responsible for preventing the invading of microbiota. Hence, the establishment of immune system in rumen epithelium is critical for the health of calves. In our study, DEPs between rumen epithelium of day 30 and day 60 significantly were enriched in NOD-like receptor signaling pathway, DEPs between rumen epithelium of day 60 and day 90 were significantly enriched in immune response, antigen processing and presentation and T-helper (Th) cell differentiation. NOD-like receptor signaling pathway can specifically sense and respond pathogen-associated molecular patterns and then secrete various cytokines by activating other signaling regulators (Liu et al., 2019). It is originally known as a critical regulator in immune responses, which specifically recognize pathogen-associated molecular patterns (Wilmanski et al., 2008). The immune system defends against pathogens and maintains the homeostasis of tissue for organism (Ganeshan and Chawla, 2014). It is suggested that CD4 T cells need to differentiate towards a Th1, Th2, Th17 to orchestrate a variety of adaptive immune responses (Butcher and Zhu, 2021). Th1 cells are primarily important in helping to mount a host defense against intracellular pathogens, whereas Th2 cells predominate in response to helminth infections and venoms (Romagnani, 1999). In addition, Th17 cells have been implicated not only in defense against opportunistic fungal or bacterial pathogens, but also in the pathogenesis of most common autoimmune diseases (Yang et al., 2014). These evidence indicated an important role of innate immune system during rumen epithelium development.

Rumen epithelium also plays roles in transporting urea, an end product of nitrogen metabolism. Urea, produced in the liver, can enter all major gastrointestinal tract compartments directly across the gastrointestinal tract wall (Hansen et al., 1976). In ruminant animals, it is transferred into the reticulo-rumen through diffusion across the rumen epithelium, where microbiota degrade recycled urea from saliva or rumen epithelium and then produce ammonia (Patra and Aschenbach, 2018). In calves, urea transporter driven urea recycling is one of the major mechanisms affecting rumen nitrogen metabolism (Stewart et al., 2005). Thus, establish the function of urea transport is of critical importance for rumen nitrogen recycle (Walpole et al., 2015). In this study, we found DEPs between day 30 and day 60 of age significantly enriched in Nitrogen metabolism and urea transmembrane transporter activity. The expression of urea transporter significantly increased on day 60 of age. These results indicated that the function of urea transport was well established after day 60 of age.

To conclude, our data indicated that keratinocyte differentiation, mitochondrion formation, the establishment of urea transport and innate immune system play central roles during rumen epithelium development. BH4 presents an important role in rumen epithelial keratinization. The biological processes of BH4 biosynthesis and molecular function of NADP binding participate in mitochondrial cristae formation. The proposed datasets provide a useful basis for future studies to better comprehend dairy calves rumen epithelial development.

## Data Availability

The datasets presented in this study can be found in online repositories. The names of the repository/repositories and accession number(s) can be found below: Data are available via ProteomeXchange with identifier PXD037396.
